# A Cell-Free Microtiter Plate Screen for Improved [FeFe] Hydrogenases

**DOI:** 10.1371/journal.pone.0010554

**Published:** 2010-05-10

**Authors:** James A. Stapleton, James R. Swartz

**Affiliations:** 1 Department of Chemical Engineering, Stanford University, Stanford, California, United States of America; 2 Department of Bioengineering, Stanford University, Stanford, California, United States of America; Center for Genomic Regulation, Spain

## Abstract

**Background:**

[FeFe] hydrogenase enzymes catalyze the production and dissociation of H_2_, a potential renewable fuel. Attempts to exploit these catalysts in engineered systems have been hindered by the biotechnologically inconvenient properties of the natural enzymes, including their extreme oxygen sensitivity. Directed evolution has been used to improve the characteristics of a range of natural catalysts, but has been largely unsuccessful for [FeFe] hydrogenases because of a lack of convenient screening platforms.

**Methodology/Principal Findings:**

Here we describe an *in vitro* screening technology for oxygen-tolerant and highly active [FeFe] hydrogenases. Despite the complexity of the protocol, we demonstrate a level of reproducibility that allows moderately improved mutants to be isolated. We have used the platform to identify a mutant of the *Chlamydomonas reinhardtii* [FeFe] hydrogenase HydA1 with a specific activity ∼4 times that of the wild-type enzyme.

**Conclusions/Significance:**

Our results demonstrate the feasibility of using the screen presented here for large-scale efforts to identify improved biocatalysts for energy applications. The system is based on our ability to activate these complex enzymes in *E. coli* cell extracts, which allows unhindered access to the protein maturation and assay environment.

## Introduction

Hydrogenase enzymes reversibly catalyze the interconversion of molecular hydrogen with protons and electrons [1]. They have drawn increasing interest from the renewable energy community [2], [3] because of their potential to become key players in biological hydrogen production schemes [4] and replacements for precious metal catalysts in fuel cells [5], [6]. Hydrogenases are classified as [NiFe], [FeFe], or [Fe] according to the composition of their active site metal cofactors. The [FeFe] hydrogenases have the highest turnover rates [1], making them attractive for energy applications, but are incompatible with many technologies due to their extreme oxygen sensitivity [7], [8]. Oxygen deactivates these hydrogenases by diffusing into the core of the protein and reacting with the catalytic iron-sulfur cofactor [9]. The development of an engineered [FeFe] hydrogenase with improved industrial traits, including decreased sensitivity to deactivation by oxygen and increased specific activity, could make the economics of biological hydrogen production technologies or fuel cells much more attractive.

Directed evolution, a laboratory technique that mimics natural evolution by selecting the fittest mutants from large libraries [10], is a promising method by which to improve the suitability of hydrogenases for various biotechnological applications [11]. However, evolving hydrogenases has proved difficult due to a lack of effective high-throughput screens. Developing a screen for improved hydrogenase mutants is challenging because hydrogenases are difficult to express in active form and because their substrate and product are difficult to measure in a high-throughput format or to link to the survival of an organism. A platform capable of supporting a large-scale screening effort has yet to be described.

Heterologous expression of [FeFe] hydrogenases became possible only recently, when the discovery of three helper proteins required for synthesis and installation of the so-called H-cluster cofactor at the catalytic site [12] enabled hydrogenase activation *in vivo* in *E. coli* [13] and *in vitro* in *E. coli* cell extracts [14], [15]. Prior to this breakthrough, the low transformation efficiencies and slow growth rates of the organisms that naturally harbor [FeFe] hydrogenases prevented screening large *in vitro*-generated gene libraries, and most directed evolution work focused on genome-wide mutagenesis to improve hydrogen production rates or oxygen tolerance of organisms that naturally produce hydrogen [16]. The ability to express mutant [FeFe] hydrogenases in *E. coli* or by cell-free protein synthesis (CFPS) has allowed the focus to shift to the enzyme itself. Instead of evolving mutant organisms that might shield their hydrogenases from oxygen or deliver reducing equivalents to the protein more effectively, mutant hydrogenases that are inherently oxygen tolerant and catalytically improved can be developed. Such mutants would retain their improvements independent of the choice of host organism, and analysis of their mutations could shed light on the relationship between [FeFe] hydrogenase structure and function.

SIMPLEX (SIngle-Molecule PCR-Linked EXpression) [17] is an entirely *in vitro* microtiter plate-based protein screening platform. *In vitro* screens and selections, which use cell-free protein synthesis to transform mutant DNA into mutant proteins, have a number of advantages over *in vivo* methods. The protein synthesis environment is open, allowing control over many aspects of the protein production and folding environment that are inaccessible *in vivo*. The protein product is synthesized within a few hours and can be assayed without a cell lysis or permeablization step. *In vitro* methods can be used to screen toxic proteins and can avoid many of the biases for which cells are notorious. While much lower in throughput than bulk methods such as ribosome display [18] and *in vitro* compartmentalization [19], the microtiter plate format allows a wide range of enzyme activities to be assessed using standard colorimetric activity assays.

**Figure 1:**
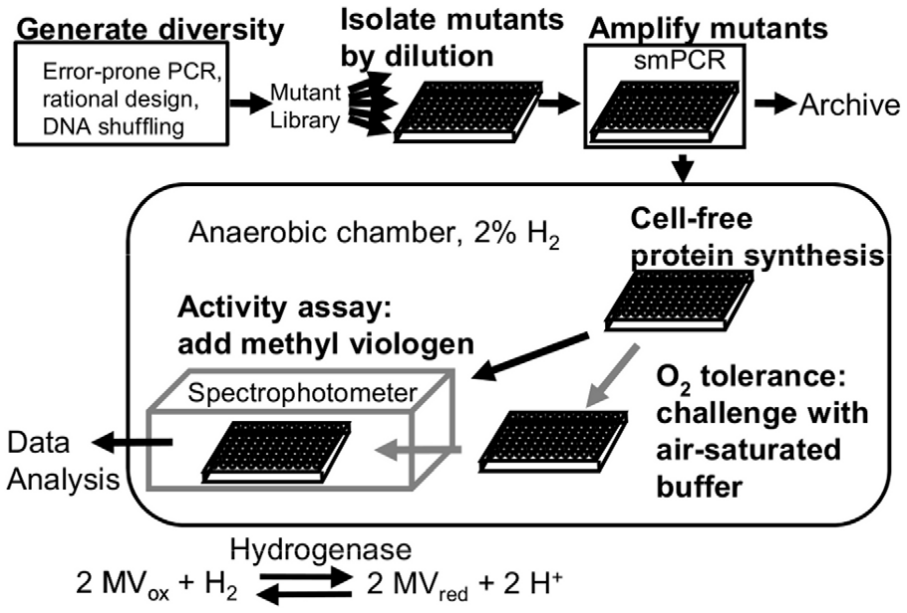
A schematic representation of the screening procedure.

Here we present a SIMPLEX-based *in vitro* screen for [FeFe] hydrogenase activity and oxygen tolerance (Figure 1), which we have used to isolate a mutant of the *Chlamydomonas reinhardtii* [FeFe] hydrogenase HydA1 with ∼4-fold improved specific activity relative to the wild-type. The system is conducive to automation and can screen on the order of 10^3^–10^4^ mutants per day.

A key step in SIMPLEX is single-molecule PCR (smPCR) [20], which amplifies mutant genes isolated by limiting dilution. This powerful technique is rapidly being adopted for use in a diverse range of fields for applications such as cancer detection [21], gene expression analysis [22], DNA methylation analysis [23], sequencing of unculturable strains [24], cell-free cloning [25], and directed evolution. We present observations regarding factors that are important for successful smPCR, which we hope will be of use to researchers attempting to establish this powerful but delicate technique in their own laboratories.

## Results and Discussion

The screen for improved hydrogenase mutants consists of mutant DNA library generation, limiting dilution, single-molecule PCR, cell-free protein synthesis, oxygen exposure, spectrophotometric measurement, and comparison to wild-type protein (Figure 1).

### Mutant DNA library generation

We chose the *hydA1* gene encoding the HydA1 hydrogenase from *C. reinhardtii* as our parent sequence, and generated a random mutant DNA library using mutagenic PCR with nucleotide analogs [26]. In general, libraries of linear mutant DNA templates may be generated by any random or rational mutagenesis method, including error-prone PCR [27], site-directed mutagenesis [28], and recombination techniques [29], [30]. The only requirements are that the resulting mutant constructs contain homoprimer annealing sequences [31], [32] to allow smPCR amplification [33] and an appropriate promoter and ribosome binding site to allow subsequent transcription and translation by the CFPS system [34]. Sequencing of a sampling of mutant genes from the library indicated a range of 2 to 10 DNA mutations per gene.

### Limiting dilution and single-molecule PCR amplification

In the SIMPLEX system, each mutant gene is isolated from the library by limiting dilution. Careful dilution to extremely low DNA concentrations allows single molecules to be distributed into the wells of a PCR plate according to Poisson distribution statistics. We added mutant library DNA at an average of 2.3 molecules per well, an amount that left approximately 10% of the wells empty, which we felt represented an optimal compromise between throughput and resolution. Decreasing the average number of genes per well leaves more wells without DNA, while increasing the average number of genes per well leads to signal distortion when two or more genes are deposited and amplified in the same well. The averaging of the signals from an improved mutant and neutral or inactive mutants in the same well raises the threshold of improvement required for detection. In addition, if the well is found to contain improved enzymes, the genes must be separated by limiting dilution or *in vivo* cloning before they can be retested and sequenced.

The isolated genes are amplified to quantities sufficient for CFPS reactions by single-molecule PCR. As the primer concentration in smPCR is very high relative to that of the single template molecule, primer-dimers and aberrant products are common. The use of a homoprimer that anneals to both ends of the target sequence has been shown to reduce primer dimerization [32] and allow single-step single-molecule PCR [35]. Since any desired primer sequence can be designed into the template, but not all sequences are effective, we used the homoprimer sequences reported by the developers of SIMPLEX (TR [36], K4 [37], and SCA2 [38]), the efficacy of which we independently confirmed.

Accurately diluting DNA more than a billion fold to the single-molecule level is not trivial. Since any template loss becomes very significant at single-molecule concentrations, we performed the dilutions in a buffered solution containing EDTA and blue dextran [17] to block nonspecific adsorption of DNA. Solutions diluted to a range of expected DNA concentrations were used as templates in a series of PCRs until a dilution was found at which only a fraction of the reactions amplified successfully. Additional amplifications were then performed to more precisely estimate the DNA concentration in that dilution by comparison of amplification frequencies with the predictions of the Poisson distribution.

Though a variety of buffers and a wide range of concentrations of magnesium, nucleotide triphosphates, and primers have been recommended by reports in the literature, we were successful using buffers supplied with commercial polymerases and Mg^2+^, dNTPs, and primer concentrations typical of standard PCR protocols. We also evaluated the addition of colloidal gold nanoparticles, which have been reported to improve PCR specificity [32]. No improvement was observed relative to reactions from which gold was omitted. The volume of each smPCR reaction was 7 µL, minimizing polymerase cost while ensuring that the liquid volume would not be significantly depleted by evaporation during the long thermocycling program. The melting step in our protocol was shortened to 10 seconds at 95°C, and even shorter times are sufficient for most targets. Minimizing the duration of this high-temperature step minimized polymerase deactivation during the high number of cycles required to amplify a single molecule to saturation. The 80-cycle program was complete in about five hours.

The sensitivity that allows amplification of a single copy of a target also makes smPCR susceptible to contamination by even very small amounts of DNA. While the specificity of the homoprimer allows smPCR to tolerate contamination by environmental or other nonspecific DNA, contamination by DNA with homoprimer annealing sites can be a serious problem. Because of this, extreme caution must be taken to avoid contaminating reagents, pipettes, surfaces, and consumables with template or product DNA. To avoid contamination, all smPCR reaction solutions were mixed in one room, and the products analyzed in a second room. Reaction components were mixed in a laminar flow hood with a dedicated set of pipettes using smPCR-only reagent aliquots, which were frequently changed. Despite these precautions, contamination was a frequent occurrence, and negative controls without template were routinely run to ensure that the reactions were contaminant-free.

Comparison of observed results with the predictions of the Poisson distribution confirmed that amplified products originated from single template molecules and allowed determination of the number of DNA molecules in the stock solution (Table 1). We prepared a mixture consisting of PCR reagents and a known volume of a highly diluted template solution. We then divided a 96-well PCR plate into thirds, and added increasing volumes of the mixture (5, 10, and 15 µL) into the wells of each section, reserving three wells for no-template controls. Within each section of the plate, observation of the fraction of the reactions in which amplification occurred allowed us to estimate the number of DNA molecules in the volume of template solution in the reaction mixture. Agreement across the sections indicated successful smPCR.

**Table 1:** Comparison of experimental results with the Poisson distribution.

Reaction size	Percent amplified	Poisson average molecules/reaction	Poisson average molecules/5 µL
5 µL	26.4	0.31	0.31
10 µL	50	0.69	0.35
15 µL	62.3	0.98	0.33

### Cell-free protein synthesis

Following amplification of isolated mutant DNA templates, smPCR plates were brought into an anaerobic chamber, and 1.5 µL volumes (containing approximately 100 ng [34], [36] of DNA) were transferred into the corresponding wells of a CFPS reaction plate without purification. The PCR plates, containing the remainder of each PCR product, were then archived at −20°C so that DNA corresponding to eventual “hits” interesting enough to merit further evaluation could be easily recovered.

Extensive modifications have been made to the PANOx-SP [39] CFPS protocol developed for general use in our laboratory to allow activation of [FeFe] hydrogenases [14]. We prepared the extracts from *E. coli* cells in which the three maturase proteins required for synthesis and activation of the [FeFe] hydrogenase active site [12] had been heterologously expressed. We conducted the CFPS reactions anaerobically to prevent deactivation of hydrogenase and its maturases. To stabilize the linear templates produced by PCR-based mutagenesis techniques, we added purified lambda phage Gam protein to the reaction mixtures to inhibit the RecBCD exonuclease complex [40] present in the extract. We also supplemented the reactions with S-adenosylmethionine (SAM), a substrate of the maturases [41]. The reactions are capable of synthesizing and maturing 40 ng/µL of active hydrogenase.

### Multiplexed hydrogenase activity assays

Methyl viologen is a redox-sensitive dye that is clear when oxidized and blue when reduced, and which can exchange electrons with [FeFe] hydrogenases. Spectrophotometric measurement of the rate of the color change as hydrogenase oxidizes H_2_ and reduces methyl viologen has been used widely in the literature as an assay for hydrogenase activity [42], and we adopted it as our primary activity assay. Preliminary hits identified with this screen could be assayed with the natural electron donor, the protein ferredoxin, in a hydrogen production assay to ensure compatibility before being carried on to the next round of mutagenesis.

We mixed the CFPS reaction products with a buffered methyl viologen solution. The absorbance at 578 nm in each well of a 96-well microtiter plate was measured over two minutes with a plate reader within the anaerobic chamber as hydrogenase consumed dissolved H_2_. The slope of the absorbance change with time was converted into a hydrogenase activity measurement using Beer's Law. The absorbance of methyl viologen solutions in this H_2_ consumption assay remained constant in the absence of added CFPS product, and the addition of negative control CFPS mixtures incubated without any DNA template or with templates encoding chloramphenicol acetyltransferase (CAT) generated slopes that were small compared to those generated by the products of CFPS reactions expressing hydrogenase.

### Multiplexed measurement of oxygen tolerance

To measure oxygen tolerance, we challenged the mutant proteins by adding a liquid solution containing dissolved oxygen. During the subsequent incubation, oxygen continually diffused out of the liquid into the anaerobic atmosphere, and was also consumed by the extract, presumably by oxygen-scavenging cytochrome oxidases in inverted membrane vesicles [43]. The oxygen concentration profile experienced by the hydrogenase mutants was therefore not constant with time and could not be quantitatively determined, but we presumed it to be the same for each reaction mixture in a given microtiter plate. For the purpose of high-throughput screening this oxygen exposure procedure proved convenient and reliable. Loss into the atmosphere and consumption by the extract eventually completely removed the dissolved oxygen, which would otherwise have interfered with measurement of the absorbance of the assay solution by oxidizing the methyl viologen.

**Figure 2:**
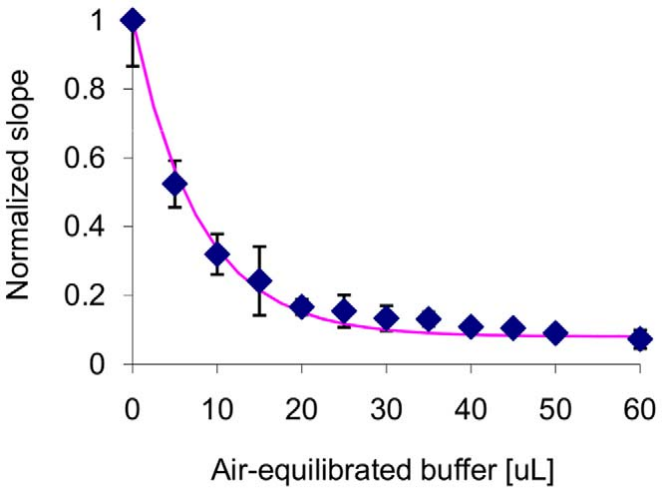
Oxygen deactivation curve for the product of CFPS reactions expressing *C. reinhardtii* HydA1. 5 µL of CFPS product were diluted with 25 µL of anaerobic Tris buffer before addition of the volume of air-saturated Tris buffer indicated on the x-axis. The slope of methyl viologen absorbance at 578 nm with time for each oxygen-exposed sample was normalized to the slope with no oxygen exposure. The pink curve represents an exponential decay fit to the data. Error bars indicate the standard deviation of triplicate measurements.

The extent of the deactivation could be controlled by adjusting the volume of oxygen-containing liquid added (Figure 2). Weak deactivation resulted in a higher signal-to-noise ratio, but led to high sensitivity to unintended variations in exposure. After very strong deactivation, the residual activity was obscured by background absorbance changes caused by factors within the cell extract. A post-exposure residual activity of 15–20% avoided both problems, generating a signal several times greater than the background observed in CAT control reactions.

The volume of CFPS reaction product used for the post-exposure activity measurement was adjusted such that the slopes of the pre- and post-exposure measurements were roughly equal and within the range that allowed accurate activity determination. For example, in the case of a 20% residual activity target, five times as much reaction product was used in the post-exposure assay as in the pre-exposure assay.

### Data processing and hit identification

The oxygen tolerance screen included two activity measurements of each CFPS reaction product with the methyl viologen activity assay, one before oxygen exposure and another after. The ratio of the two measurements was used as a measure of oxygen tolerance and was the criterion by which hits were identified. The pre-exposure test allowed the oxygen tolerance score of each mutant to be normalized by initial activity, eliminating the influence of variation in expression across CFPS reactions. In addition, a minimum pre-exposure activity cutoff was established. Mutant hydrogenases with pre-exposure activities below this threshold were disqualified, since the low signal-to-noise ratio in these wells inflated the residual activity ratio.

**Figure 3:**
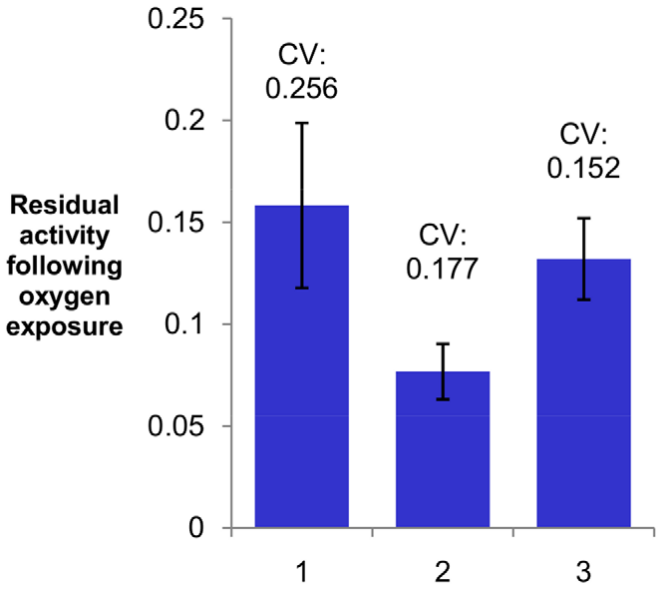
Assessment of the well-to-well error of the screen in three sets of identically performed 10 µL CFPS reactions. Residual activity following oxygen exposure is represented by the average pre-exposure/post-exposure activity ratio. Error bars indicate the standard deviation for n = 88, 72, and 71 wells respectively. The coefficient of variance is given above each bar. 1 and 5 µL of CFPS product were assayed in the pre-exposure and post-exposure measurements, respectively, and the post-exposure activity was normalized to 1 µL. 15 µL of O_2_-equilibrated buffer were used for deactivation. *C. reinhardtii* HydA1 was expressed in Experiments 1 and 2, and *C. pasteurianum* CpI was expressed in Experiment 3. The difference in average residual activity between Experiments 1 and 2 is likely due to differences in the extent of oxygen diffusion out of the aerobic buffer before addition to the hydrogenase solutions, and highlights the need for wild-type controls on each plate.

The activity and residual activity ratio of each mutant were compared to those of wild-type hydrogenase controls. Performing wild-type control reactions in each microtiter plate enabled us to account for plate-to-plate and day-to-day variations in CFPS reaction performance and oxygen exposure effectiveness. The ability of a screen to identify an improved mutant is inversely related to the standard deviation of measurements of identically prepared wild-type reactions. We were able achieve a coefficient of variance of measurements of oxygen tolerance of ∼15–25%. The precision of repeated measurements of oxygen tolerance is shown in Figure 3. Experiments 1 and 2 measured the error of residual activity measurements on the products of identically performed CFPS reactions expressing wild-type *C. reinhardtii* HydA1. In Experiment 3 wild-type *C. pasteurianum hydA* was used as the template. The measured error is in a range typical of microtiter plate screens. If a hit threshold were set two standard deviations above the mean, the screen could identify mutants improved in oxygen tolerance by ∼30–50%.

### Isolation and characterization of a mutant with improved specific activity

We used this method to screen ∼30,000 *hydA1* mutants (an average of 2 mutants per well of 150 96-well plates). We found no hydrogenase mutants that were significantly more oxygen-tolerant than the wild-type. However, pre-exposure hydrogen consumption activity measurements of several mutants were significantly higher than wild-type HydA1 activity measurements, and these mutants were selected for further evaluation. The DNA templates were recovered from archived PCR plates, re-amplified by PCR, and evaluated by expression in plate-based CFPS followed by activity quantification of the protein products with the methyl viologen activity assay.

To measure the specific activity of the most improved mutant, we added the radioactive amino acid L-[U–^14^C]-leucine to the CFPS mixtures, and measured its incorporation into the protein product by TCA precipitation and liquid scintillation counting [44]. We intentionally limited the protein yields in these reactions to promote complete activation, and their proximity to the sensitivity limit of our protein quantification technique led to a significant standard deviation among the measurements. We calculated the specific activity in the hydrogen production direction after measuring hydrogen produced over time by CFPS product mixed with sodium dithionite-reduced methyl viologen using an H_2_ analyzer [14].

**Figure 4:**
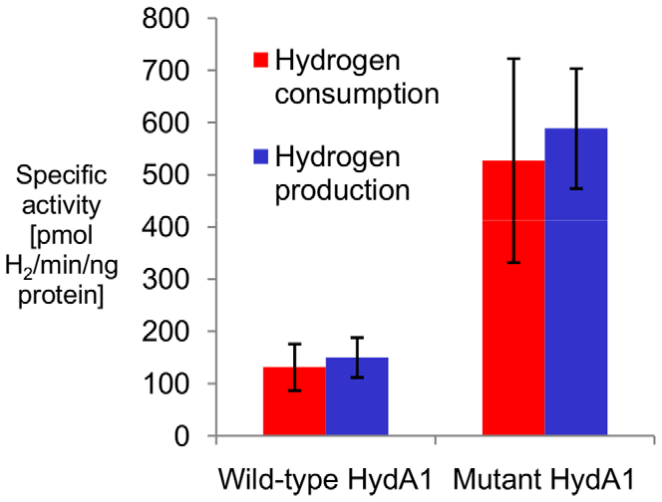
Specific activities of wild-type and mutant *C. reinhardtii* HydA1. Specific activity was calculated for each using cell-free protein synthesis products in the hydrogen consumption direction (blue bars) and the hydrogen production direction (red bars). Error bars indicate the standard deviation of at least n = 4 independent experiments.

The most improved HydA1 mutant is more active than the wild-type by a factor of ∼4 (Figure 4). Interestingly, the activity of the mutant seems to have increased by the same amount in both directions, indicating that its mutations do not bias its reversibility [45]. The specific activity of the wild-type HydA1 is 150 pmol H_2_/min/ng protein. This value is ∼20% of typical activities reported for HydA1 purified after production in hosts naturally possessing [FeFe] hydrogenases [46]–[49], but very similar to specific activities reported for protein produced *in vivo* in *E. coli* [13]. Because of this discrepancy, we cannot rule out the possibility that the isolated mutant is improved in its ability to be activated in the CFPS system rather than in its turnover rate. To confirm the improvement, future work will include expression of the mutant in a natural [FeFe] hydrogenase host in place of the wild-type gene.

DNA sequencing revealed two amino acid mutations: G172D and N267S (residue numbers include the 55 amino acid targeting sequence, as in GenBank accession AY055755.1). A multiple sequence alignment generated by ClustalW (Figure S1) showed that both of these amino acids are immediately adjacent in the primary sequence to residues that are highly conserved among [FeFe] hydrogenases. Interestingly, both are non-conservative changes. No crystal structure is available for *C. reinhardtii* HydA1, but the position corresponding to G172 in the homologous hydrogenase CpI from *Clostridium pasteurianum* [50] is in an alpha helix approximately 8Å from the active site H-cluster, while the residue corresponding to N267 is on an external loop.

This is the first improved [FeFe] hydrogenase to be isolated by directed evolution. Previously, a mutant of the large subunit of the *E. coli* [NiFe] hydrogenase 3 was found that improved the hydrogen production capability of its host cell [51]. This membrane-bound hydrogenase could not be assayed in purified form. The mutant was identified with an *in vivo* screen using chemochromic hydrogen detectors, with a throughput theoretically comparable to that of the screen presented here. Recent progress in the *in vivo* expression of [FeFe] hydrogenases in *E. coli* could allow this organism to be used as a host for *in vivo* screening of heterologous [FeFe] hydrogenases using a similar system.

Although we developed the screen to search for oxygen-tolerant mutants, it can also be used to identify mutants with increased specific activity, as well as mutants improved in any other activity-related trait. For example, thermostable mutants could be identified by incubating the CFPS products at high temperatures and then measuring residual activity. Since the screen is based on cell-free protein synthesis of [FeFe] hydrogenase mutants, the experimenter has unfettered access to the protein synthesis, maturation, and activity assay environment, providing flexibility in devising screening conditions that would not be possible in traditional *in vivo* platforms. The cell-free nature of the screen may prove especially advantageous when screening for characteristics beneficial for *in vitro* applications such as fuel cells.

Our results further validate SIMPLEX as a valuable technique in the repertoire of directed evolution methods. The HydA1 hydrogenase from *C. reinhardtii* is a difficult target for both smPCR, because of the 60% GC content of the gene, and CFPS, because of the complexity of the protein. The success of SIMPLEX in evolving this protein validates the system for use evolving other complex, industrially-relevant enzymes.

The ability of our screen to identify mutants with improved specific activities, while simultaneously failing to identify mutants with improved oxygen tolerance, hints at the relative frequency of the two phenotypes in the mutant library. It is possible that any improvement in oxygen tolerance will require multiple simultaneous mutations to close off all of the routes by which oxygen can reach the active cluster. If this is the case, either new screens with higher throughputs or a massive effort with a screen such as the one presented here will be required, along with rational or semi-rational library design [52] to restrict the sequence space to be searched.

## Materials and Methods

### Template preparation, purification, and dilution

DNA templates for smPCR were linear cell-free protein synthesis expression templates consisting of a codon-optimized gene encoding *C. reinhardtii* HydA1 hydrogenase with a T7 RNA polymerase promoter and terminator and an *E. coli* ribosome binding site [14]. The 55 amino-acid targeting sequence following the N-terminal methionine was removed, and the first nine codons of the chloramphenicol acetyltransferase gene, which has been shown to express well in the CFPS system, were inserted after the start codon to ensure accessibility of the RBS on the mRNA transcript. The templates were amplified from a plasmid using primers that annealed to sequences on the plasmid outside the expression region, and included 5′ regions that extended the homoprimer annealing sequence of choice onto each end of the linear product. Nucleotide analog mutagenesis [26] was used to generate mutant libraries.

Templates were purified with QiaQuick PCR cleanup kits (Qiagen, Valencia, CA), by purification from Novex TBE polyacrylamide gels (Invitrogen, Carlsbad, CA), or by phenyl chloroform extraction and ethanol precipitation.

DNA was quantified by measuring absorption at 260 nm with a spectrophotometer. Desired dilution levels were calculated based on the calculated molecular weight of the template molecules (based on an average of 660 Da/bp). DNA was serially diluted into TE buffer with 0.1% (w/v) blue dextran, usually in steps of 1 µL into 1 mL. Tubes were thoroughly vortexed between dilution steps. DNA stocks were diluted to an estimated concentration of 100 molecules/µL for use in SIMPLEX screens. The concentrations of working stocks were confirmed by Poisson distribution experiments as described, and the amount added to the smPCR mixture was adjusted as necessary to achieve the desired number of molecules per well of a PCR plate.

### Single-molecule PCR

Single-molecule PCR reaction mixtures consisted of 1X manufacturer's PCR buffer (supplemented with magnesium chloride to 2 mM when necessary); 0.2 mM of each of the four deoxyribonucleotides; 0.5 µM of the homoprimer; either 0.02 units/µL Platinum Taq (Stratagene, La Jolla, CA), 0.03 units/µL Pfu Turbo (Stratagene, La Jolla, CA), 0.03 units/µL Pfu Ultra (Stratagene, La Jolla, CA), or 0.02 units/µL Phusion (Finnzymes, Espoo, Finland); and template DNA. Reaction volumes were typically 7 µL. Reactions were prepared in a laminar flow hood (Nuaire, Plymouth, MN) to prevent contamination.

Reactions were incubated at 95°C for one minute to activate the hot-start polymerase and denature the template, then thermocycled 80 times through 10 seconds at 95°C, 20 seconds at 55°C, and an extension temperature and time recommended by the manufacturer (generally 72°C for 1 minute/kb of template length). When Phusion polymerase was used, the denaturation step was 5 seconds at 98°C, the annealing temperature was 62°C, and the extension time was 15 seconds/kilobase.

### Cell-free protein synthesis

Extracts were prepared anaerobically from *E. coli* BL21 cultures expressing the three helper proteins required for maturation of [FeFe] hydrogenases [14]. Cells were grown aerobically on a defined medium [14] at 37°C in a fermenter to OD ∼6–8. The culture was then made anaerobic by switching the gas feed from air to argon. After 45 minutes, the temperature was dropped to 15°C, and expression of the helper proteins was induced by addition of 0.5 mM IPTG. At this point the medium was also supplemented with 0.33 mg/mL ferric ammonium citrate and 10 mM fumarate. After 16 hours the cells were harvested, pelleted, washed, and homogenized, and the lysate centrifuged and frozen, as described previously [14] except that all the steps were performed under aerobic conditions. Before use in CFPS reactions, the extract was incubated at room temperature with 1 mM ferrous ammonium sulfate and 1 mM sodium sulfide for 1–2 hours [14].

CFPS reactions were conducted in 384-well PCR plates. 7.5 µL reaction mixtures were prepared by adding 3 µL of Mix 1 to a well, then 1.5 µL of crude smPCR product, then 3 µL of Mix 2. Mix 1 consisted of small molecule cofactors (listed in Table S1 at their concentrations in the final CFPS mixture). Mix 2 consisted of the following, given with their concentrations in the final CFPS mixture: 0.1 mg/mL T7 RNA polymerase (overexpressed in *E. coli* and purified in house), 6.7 µg/mL lambda phage Gam protein (expressed in CFPS and purified in house), 0.01% 31R1 antifoam (Sigma Aldrich, St Louis, MO), and 25% v/v cell extract. ^14^C-leucine was omitted in the high-throughput screen reactions but included at 5.25 µM when characterizing hits to allow quantification of synthesized protein by scintillation counting of incorporated radiation [44]. Reactions were incubated for between six and 16 hours at room temperature in an anaerobic glove box (Coy Laboratory Products, Grass Lake, MI) before being assayed.

### Methyl viologen activity assay

Activity of the hydrogenase produced in the CFPS reactions was obtained by measuring the rate of change of the absorbance at 578 nm of 200 µL of a 2 mM solution of methyl viologen in 50 mM Tris buffer at pH 8 with a VersaMax plate reader (Molecular Devices, Sunnyvale, CA) following addition of CFPS product. Immediately before the assay was conducted, the methyl viologen solution was reduced by addition of titanium citrate until a light blue color persisted. Absorbance was monitored for two minutes at room temperature in 96-well flat-bottom plates. Slopes were converted into activity using Beer's Law with an extinction coefficient of 9.78 AU/min/mM.

### Oxygen exposure

50 mM Tris buffer at pH 8 was stored outside the glove box and allowed to come to equilibrium with oxygen, so that the dissolved oxygen concentration was expected to be ∼0.25 mM. A small vial was filled to the brim with this solution to minimize headspace, sealed, and moved into the anaerobic chamber immediately before the oxygen exposure step in the protocol. The vial was opened and the buffer poured into a trough, from which it was aspirated by a multichannel pipetter or the multichannel head of a liquid handler. A volume of buffer expected to deactivate the CFPS product to the desired extent was dispensed into each well of the oxygen-exposure plate. The plate was incubated for 10 minutes, after which time methyl viologen could be added to assay residual activity. No decrease in blue color was noticeable upon methyl viologen addition, indicating that after 10 minutes all the oxygen had either been consumed by the extract or had diffused into the atmosphere of the anaerobic chamber.

### High-throughput activity measurements

A liquid handling machine (EpMotion 5070, Eppendorf North America, Westbury, NY) first pipetted 72 µL of anaerobic Tris buffer (50 mM, pH 8) into each well of eight 96-well spectrophotometer plates, four for pre-oxygen exposure and four for post-oxygen exposure activity measurements of the mutant proteins in one 384-well CFPS plate. 6 µL of CFPS product were transferred from the 384-well CFPS reaction plate into the wells of four of the 96-well plates, and mixed with the buffer by pipetting up and down five times. 13 µL were then transferred from these wells to the corresponding wells of the other four plates, and again mixed. These plates, which would be used for the pre-oxygen exposure measurements, thus contained 1/5 as much CFPS product as did the first set of plates, which would be used for the post-oxygen exposure measurements. After the liquid handler dispensed 20 µL of air-equilibrated buffer into each well of the post-deactivation plates, the wells of all eight plates contained 85 µL of liquid. 115 µL of methyl viologen solution was then added to each well to give a final concentration of 2 mM, and the plate was immediately inserted into the spectrophotometer.

### Hit confirmation and characterization

Genes were reamplified by PCR and re-tested in replicate CFPS reactions. Radioactive leucine was added to allow quantification of protein production by TCA precipitation and liquid scintillation counting [44]. Specific activity in the hydrogen consumption direction was calculated by dividing the hydrogen consumption rate determined from the methyl viologen assay data by the soluble protein yield determined by measurement of incorporated radioactive leucine. Mutants were tested for hydrogen production ability using a Peak Performer hydrogen analyzer (Peak Laboratories, Mountain View, CA) as previously reported [14]. Crude CFPS solution containing 10–40 ng of hydrogenase was added to 1 mL of a solution consisting of 5 mM methyl viologen and 25 mM sodium dithionite in 50 mM Tris-HCl pH 6.8 in a sealed vial. The headspace was exchanged with nitrogen, and 100 µL samples were removed from the headspace with a syringe and injected into the hydrogen analyzer. Specific activity in the hydrogen production direction was calculated by dividing the hydrogen production rate calculated from the hydrogen analyzer data by the soluble protein yield calculated by measurement of incorporated radioactive leucine. Due to the observed dependence of the percentage of hydrogenase that is matured on the expression level, specific activities were only compared when the expression levels of each sample were similar.

For molecular modeling, we used the PyMOL Molecular Graphics System with PDB entry 1feh.

## Supporting Information

Figure S1 Multiple sequence alignment of representative [FeFe] hydrogenases. Asterisks, colons, and periods indicate locations with homology across different species. Red dots indicate the locations of the mutations identified in this study. Both mutations are adjacent to highly conserved residues. CpI: HydA from *Clostridium pasteurianum*. C.r.HydA1 and C.r.HydA2: HydA1 and HydA2 from *Chlamydomonas reinhardtii*.
(2.64 MB TIF)

Table S1 Final concentrations of small molecule components of the CFPS mixture.
(0.04 MB DOC)
