# Supplementary figures and images for: A Cell-Free Microtiter Plate Screen for Improved [FeFe] Hydrogenases
Source: PLoS One. 2010 May 10;5(5):e10554. doi: 10.1371/journal.pone.0010554 (PMC2866662; doi:10.1371/journal.pone.0010554)

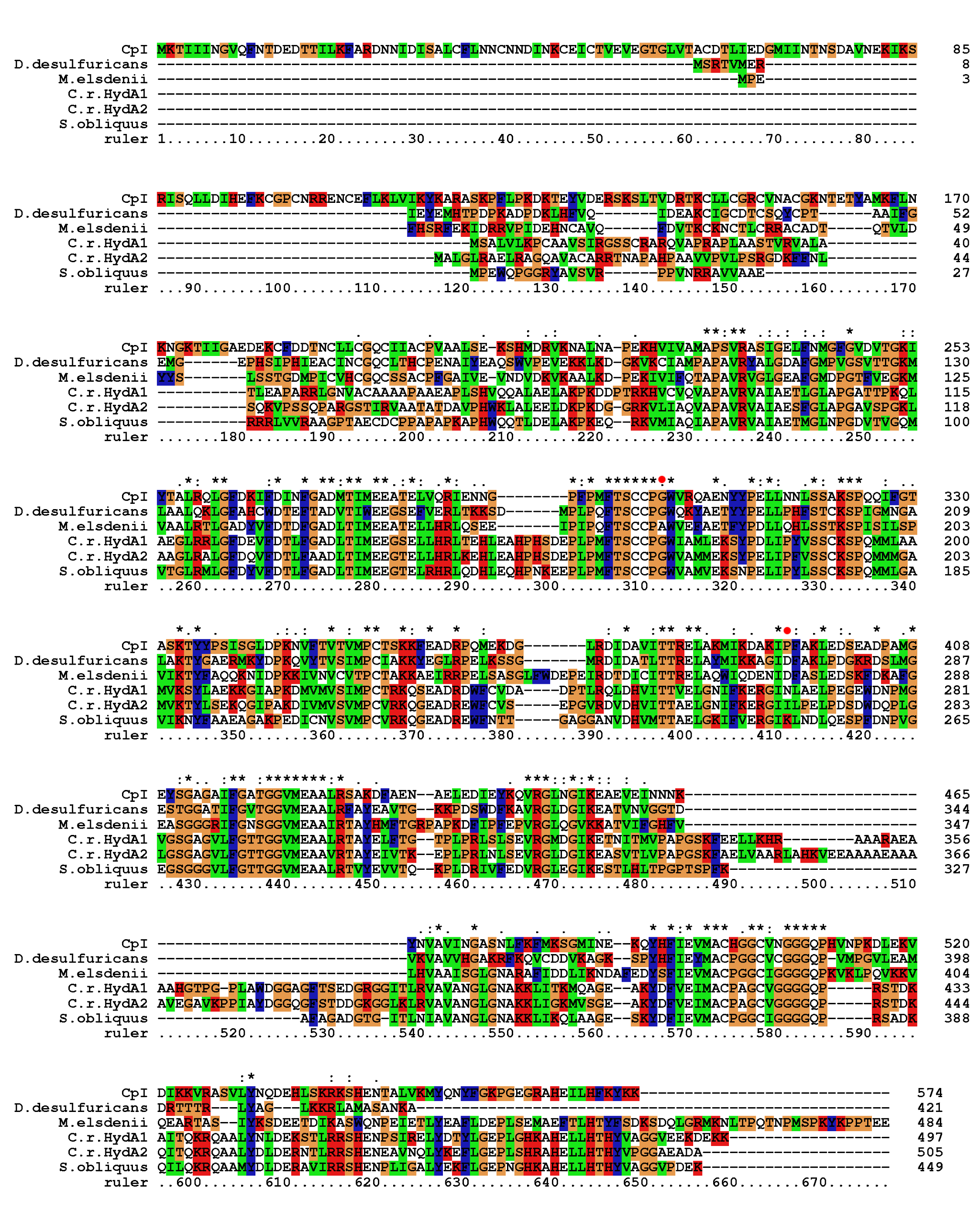

Supplement: Figure S1 — Multiple sequence alignment of representative [FeFe] hydrogenases. Asterisks, colons, and periods indicate locations with homology across different species. Red dots indicate the locations of the mutations identified in this study. Both mutations are adjacent to highly conserved residues. CpI: HydA from Clostridium pasteurianum. C.r.HydA1 and C.r.HydA2: HydA1 and HydA2 from Chlamydomonas reinhardtii. (2.64 MB TIF) [file pone.0010554.s001.tif]
