# Supplementary material for: A Cell-Free Microtiter Plate Screen for Improved [FeFe] Hydrogenases
Source: PLoS One. 2010 May 10;5(5):e10554. doi: 10.1371/journal.pone.0010554 (PMC2866662; doi:10.1371/journal.pone.0010554)
Supplement: Table S1 — Final concentrations of small molecule components of the CFPS mixture. (0.04 MB DOC) [file pone.0010554.s002.doc]

**Table S1:** Final concentrations of small molecule components of the CFPS mixture

| Mg(Glu)_2_ | 10 | mM |
| --- | --- | --- |
| NH_4_(Glu) | 10 | mM |
| K(Glu) | 175 | mM |
| ATP | 1.25 | mM |
| GTP | 1.00 | mM |
| UTP | 1.00 | mM |
| CTP | 1.00 | mM |
| Folinic acid | 34 | µg/mL |
| *E. coli* tRNA mix | 170.6 | µg/mL |
| 20 amino acids (each) | 2.00 | mM |
| PEP | 30.00 | mM |
| NAD | 0.33 | mM |
| CoA | 0.27 | mM |
| Oxalic acid | 2.70 | mM |
| Putrescine | 1.00 | mM |
| Spermidine | 1.50 | mM |
| Fe(NH_4_)_2_(SO_4_)_2_ | 0.80 | mM |
| Na_2_S | 0.80 | mM |
| S-adenosylmethionine | 1.00 | mM |

SHAP SHAPE
